# Proteomic Analysis of Aphid-Resistant and -Sensitive Rose (*Rosa Hybrida*) Cultivars at Two Developmental Stages

**DOI:** 10.3390/proteomes6020025

**Published:** 2018-05-25

**Authors:** Sowbiya Muneer, Hai Kyoung Jeong, Yoo Gyeong Park, Byoung Ryong Jeong

**Affiliations:** 1 Department of Horticulture, Division of Applied Life Science (BK21 Plus), Gyeongsang National University, Jinju 660-701, Korea; sowbiya.muneer@vit.ac.in (S.M.); jhksmile@naver.com (H.K.J.); iuyiuy09@naver.com (Y.G.P.); 2 Centre for Agricultural Innovations and Advanced Learning, Vellore Institute of Technology [VAIAL], Vellore 632014, Tamil Nadu, India; 3 Institute of Agriculture and Life Science, Gyeongsang National University, Jinju 660-701, Korea; 4 Research Institute of Life Science, Gyeongsang National University, Jinju 660-701, Korea

**Keywords:** aphids, proteomics, cut rose, resistant, sensitive

## Abstract

The rose is one the most commercially grown and costly ornamental plants because of its aesthetic beauty and aroma. A large number of pests attack its buds, flowers, leaves, and stem at every growing stage due to its high sugar content. The most common pest on roses are aphids which are considered to be the major cause for product loss. Aphid infestations lead to major changes in rose plants, such as large and irregular holes in petals, intact leaves and devouring tissues. It is hypothesized that different cut rose cultivars would have different levels of sensitivity or resistance to aphids, since different levels of infestation are observed in commercially cut rose production greenhouses. The present work compared four cut rose cultivars which were bred in Korea and were either resistant or sensitive to aphid infestation at different flower developmental stages. An integrative study was conducted using comprehensive proteome analyses. Proteins related to ubiquitin metabolism and the stress response were differentially expressed due to aphid infestation. The regulations and possible functions of identified proteins are presented in detail. The differential expressions of the identified proteins were validated by immunoblotting and blue native page. In addition, total sugar and carbohydrate content were also observed.

## 1. Introduction

Roses (*Rosa hybrida*) are used for ornamental purposes, essential oil production, cosmetics, and for the preparation of traditional jams (such as Gulkand or Murabba) from their petals. Roses were cultivated and domesticated in early 3000 BC such as Asia, Europe, and the Middle East. About 30,000–35,000 rose cultivars are bred throughout the world [1,2]. Several studies on genetic mapping reveal the base and augmentation of their petals, their different colors, and important volatile compounds for the preparation of perfumes or scents [3,4,5,6,7,8]. Unfortunately, roses have become prey for many pests due to their high carbohydrate and sugar content. Among various pests, aphids are the most common predator on them and destroy their aesthetic beauty and value.

Aphids are small temperate climate insects and feed themselves on translocating elements of sieve tubes in the phloem [9]. Their entry into the plants is through epidermis and mesophyll with their stylet-like mouthparts [10]. During their entry into sieve tube of phloem, they secrete saliva which contains peroxides, β-glucosidases, and other signal generating enzymes [11]. These signals frequently stimulate the host to generate reactive oxygen species (ROS), leading to intracellular oxidative damage [12,13,14,15,16,17]. In previous studies, the relationship between the host plant and aphid have shown variation towards ROS generation and their scavenging activity [17,18,19,20,21]. In other studies, it has been observed that host plant activates hormonal pathways particularly jasmonic acid (JA) and salicylic acid (SA) for defense response to aphid attack [9,18,22].

Aphids on roses can be controlled by several methods which include air circulation, practice sanitation, some chemical sprays, and cultivars with a good resistance to major aphid attacks or other diseases [23]. Conversely, the signs and symptoms of aphids vary according to the host plant species, types of aphids or the combination of both [24]. The interaction of aphids and their host has been studied at a transcriptional level, while, proteomic data is available only for few crops like wheat and tomato [25,26]. There is no study on the interaction between aphid-resistant and/or -sensitive plants. Thus, the aim of this research was to use a differential proteomic analysis to identify a putative relationship between aphid-resistant and/or aphid-sensitive cultivar for possible defense mechanism in four cultivars of rose var. ‘Stella’ and ‘Alibaba’ as aphid-resistant cultivars ‘Sun star’ and ‘Haetsal’ as aphid-sensitive cultivars. For proteomic analysis, we evaluated first dimensional SDS-PAGE and MALDI-TOF-TOF to identify proteins involved in aphid-resistant and tolerance defense mechanism followed by immunoblot analysis. In addition to immunoblotting, we validated the identified proteins related to photosynthesis by traditional first dimension blue native polyacrylamide gel electrophoresis (BN-PAGE). Besides, total sugar and carbohydrate content were also analyzed. The information obtained at the proteome level in this study can serve as a tool to decipher molecular mechanism underlying aphid-resistant and -sensitive rose cultivars. 

## 2. Material and Methods

### 2.1. Sample Collection and Transportation

**Figure 1:**
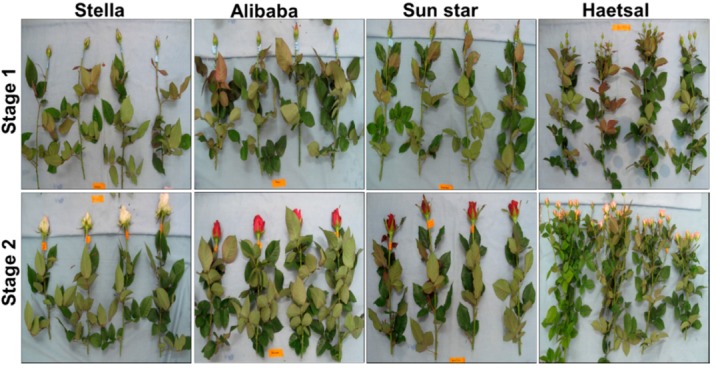
Representation/photographs of cut roses used for experiments-resistant and/or -sensitive to aphids. Aphid-resistant cultivars are ‘Stella’ and ‘Alibaba’; Aphid-sensitive cultivars are ‘Sun star’ and ‘Haetsal’. Stage 1 indicates roses at developed bud stage and stage 2 indicates roses at developed flower stage.

Aphid-infested rose cuttings were procured from Gimhae local rose farm, South Gyeongsang province, Republic of Korea (Figure 1). In this study both resistant (Stella and Alibaba), and sensitive (Sun star, and Haetsal) cultivars were compared to their suitability for breeding. All of the rose cultivars were collected at the bud and flower stages; these were transported in water wrapped in newspaper to prevent dehydration and mechanical injury, to the laboratory, Department of Horticulture, Gyeongsang National University. After transportation resident aphids were removed, the leaves from all cultivars were carefully picked and immediately frozen in liquid nitrogen followed by grinding in chilled pestle and mortar to fine powder, stored in −80 °C until use. 

### 2.2. Protein Extraction and First Dimensional Gel Electrophoresis

Protein extraction was done according to our previous methods [27]. The frozen powdered samples from all cut rose cultivars was extracted with extraction buffer (pH 7.5) containing 40 mM (*w*/*v*) Tris-HCl, pH 7.5, 2 mM (*w*/*v*) EDTA, 0.07% (*w*/*v*) β-mercaptoethanol, 2% (*w*/*v*) PVP and 1% (*v*/*v*) Triton X-100. The extract was centrifuged at 13,000 rpm for 10 min at 4 °C. The supernatant was mixed with protein-dye and 20 μg proteins were loaded on 12.5% polyacrylamide gel on PROTEAN II (Bio-Rad, Hercules, CA, USA). The protein concentration was determined by the Bradford method using BSA (bovine serum albumin) as a standard curve. After electrophoresis, the gels were stained with a commercial available silver stain according to manufacturer’s instructions (Bio-Rad, Hercules, CA, USA).

### 2.3. Protein in Gel Digestion

The protein bands on 1DE gels were excised manually with a clean razor blade and were chopped into small pieces. The excised bands were transferred to 0.5 mL clean eppendorf tubes and was proceeded for in gel digestion according to our previous methods [27]. The gel pieces were destained with freshly-prepared 30 µL of a 1:1 (*v*/*v*) mixture of the two destaining reagents K_3_[Fe(CN)_6_] (potassium ferricyanide) and Na_2_S_2_O_3_ (sodium thiosulphate pentahydrate) by incubating for 30 min at room temperature (25 °C) with gentle agitation. The destaining solution was removed and gel particles were washed with distilled water and 50 mM NH_4_HCO_3_/ACN (*v*/*v*) (ammonium bicarbonate/acetonitrile) for 15 min (1:1). The gel particles were then covered again with ACN (acetonitrile) for 2–5 min and were dried in a vacuum centrifuge. After drying, the gel particles were rehydrated in 10 mM dithiothreitol/50 mM NH_4_HCO_3_ (ammonium bicarbonate) (1:1) by incubating at 56 °C for 45 min. The Eppendorf tubes containing gel particles were cooled to room temperature (25 °C) in dark conditions and a rehydrated solution was removed. The gel particles were again washed with 50 mM NH_4_HCO_3_ (ammonium bicarbonate) and ACN (acetonitrile) (1:1) with one or two changes for 15 min per change. The gel particles were covered with ACN (1:1) (acetonitrile) to shrink the gel pieces and then dried in a vacuum centrifuge. After washing, the gel particles were treated with freshly prepared 5 ng of trypsin (Sigma-Aldrich, St. Louis, MO, USA) prepared in 1 M HCl and was incubated overnight at 37 °C to keep gel. After overnight incubation the centrifuge tubes containing gel particles were spun down and resulting supernatants (peptide mixtures) were collected in new centrifuge tubes. The resulting peptides were vacuum dried and dried peptides were dissolved in a 3–5 µL of sample solution containing 50% ACN (acetonitrile) and 0.1% TFA (trifluoroacetic acid). The solutions were stored at −20 °C until further use. 

### 2.4. Protein Identification Using MALDI-TOF MS and MS/MS Analysis

The digested peptide solution was spotted onto the MALDI-TOF MS target plate with a pipette. MALDI-MS analysis was performed with a Voyager DE-STR mass spectrometer (Applied Biosystems, Framingham, MA, USA) [27]. A two-point internal standard [des-Arg1-Bradykinin (*m/z* 904.4681) and neurotensin (*m/z* 1672.9175)] was used for calibration. The software Data Explorer (Perspective Biosystems, Inc., Framingham, MA, USA; v5.0) was used to view and process data files. The peptide mass fingerprint (PMFs) obtained from each digested protein were compared with PMFs in the non-redundant National Center for Biotechnology Information database (NCBInr, 2011/02/01, entries from all green plants) using the MASCOT database (http://www.matrixscience.com). An ABI 4800 Plus TOF–TOF Mass Spectrometer (Applied Biosystems, Framingham, MA, USA) was employed for MS and MS/MS analyses of the peptides. The instrument was set at 200 Hz ND: 355 nm YAG laser operations. Signal/noise ratios >25 (1:1) and the ten with higher intense ions were used to following MS/MS analysis in 1 kV mode, 1000–1250 consecutive laser exposure. The MS and MS/MS spectra data were analyzed using NCBI and Protein Pilot V.3.0 database software (with the MASCOT V.2.3.02 database search engine) at 50 ppm of mass tolerance. Oxidation of methionines and carbamidomethylation of cysteines were allowed for the MS/MS spectra search in the databases. Individual peptide ion scores were searched using a statistically significant threshold value of *p* = 0.05. The identified proteins were classified into different categories of biological processes in which they are involved according to gene ontology (http://www.geneontology.org/).

### 2.5. Immunoblot Analysis

For the immunoblot analysis the same protein extraction was followed as for first dimensional gel electrophoresis (protocol given above). After electrophoresis, the gels were transferred to 0.45 µM nitrocellulose membrane (Sigma-Aldrich). After protein transfer, the blots were blocked with 5 % nonfat dry skimmed milk or bovine serum albumin (Sigma-Aldrich). After washing with TBS, the blots were incubated with monoclonal primary antibodies 1:1000 dilution of anti-SOD (Cell Signaling #2770, Danvers, MA, USA) for superoxide dismutase, and 1:1000 dilution of anti-APX/L (Cell signaling #AS08 368) for ascorbate peroxidase overnight at 4 °C. The blots were treated with 1:1000 dilution HRP-linked anti-rabbit 1gG (Cell Signaling #7074) for 1 h as a secondary antibody. Chemiluminescence reactions were performed with super signal west pico chemiluminescent substrates (Cell Signaling SignalFire^TM^ ECL Reagent #6883) and images were captured on ChemiDoc imaging system (BioRad, Hercules, CA, USA).

### 2.6. First Dimensional Blue Native Page (BN-PAGE)

For blue native polyacrylamide gel electrophoresis (BN-PAGE), 5 g of leaf samples from each rose cultivar were used for chloroplast/thylakoid extraction. The extraction of chloroplast/thylakoid was followed according to our recent methodology [28]. Approximately 5 g of leaves were homogenized in liquid nitrogen in ice cold B1 buffer containing 330 mM sorbitol/50 mM HEPES/5 mM MgCl_2_/2 mM EDTA/2 mM NaF, pH 7.8 at 4 °C. The thick homogenate was filtered through three layers of miracloth (Calbiochem, San Diego, CA, USA) and centrifuged at 4500 rpm for 10 min (4 °C). The resulting protein pellet were re-suspended in ice cold B2 buffer containing 20 mM tricine, 70 mM sucrose and 5 mM MgCl_2_, pH 7.8 and were re-centrifuged same as in first step. The resulting protein pellet were washed 3–4 times with washing buffer containing 330 mM sorbitol, 50 mM BisTris-HCl (pH 7.0), and 0.1 mg mL^−1^ pefabloc and finally re-suspended in 2% *w*/*v* n-dodecyl-β-d-maltoside (Sigma-Aldrich, St. Louis, MO, USA). The chloroplast/thylakoids proteins were then solubilized in buffer and loading dye (5% CBB-G250, 100 mM BisTris-HCl, pH 7.0, 30% *w*/*v* sucrose and 500 mM ε-amino-n-caproic acid). The samples were then loaded on 5–12% *w*/*v* native polyacrylamide gel after protein quantification by Bradford method. The electrophoresis was performed at 4 °C in a Protean II xi cell electrophoresis system (Bio-Rad, Hercules, CA, USA) with following voltage: 60 V for 30 min; 100 V for 1 h; and 150 V continuous until all proteins bands became visible. 

### 2.7. Sugar and Carbohydrate Content

Sugar content was estimated according to the methodology given by Chow and Landhäusser [29]. Approximately 100 mg of dry samples were suspended in 10 mL of 95% ethanol followed by incubation at 65 °C for 1 h. After incubation, the volume was made up to 25 mL by adding ethanol (95%). About 1 mL of aliquot was mixed with 5% aqueous phenol (Sigma Aldrich, St. Louis, MO, USA) and sulfuric acid (H_2_SO_4_) (Sigma Aldrich, St. Louis, MO, USA) and absorbance was recorded at 485nm using spectrophotometer. The corresponding concentration was determined against a standard curve prepared by using glucose solution.

Carbohydrate was estimated by the anthrone method with minor modifications [30]. About 100 mg of dry samples were hydrolyzed in water bath with 2.5 N hydrochloride for 3 h. The samples were thereafter concentrated with solid sodium carbonate till effervescence stopped. The samples were then centrifuged at 10,000 rpm for 10–15 min and supernatant were used for analysis with 4 mL of anthrone reagent (Sigma-Aldrich, St. Louis, MO, USA). The samples were heated in water bath for 8–10 min and cooled down till the color of the samples changed from green to dark green and absorbance was taken at 630 nm using spectrophotometer. The concentration of carbohydrate was measured according the standard curve prepared by using glucose solution.

### 2.8. Statistical Analysis

A complete randomized design was utilized with four replicates. Differences were considered significant if *P*-values were under 0.05, and means were compared by Tukey’s test using SPSS program version 12.0 (SPSS Inc., Chicago, IL, USA). 

## 3. Results and Discussion

### 3.1. Relative Proteome Changes and Protein Identification

**Figure 2:**
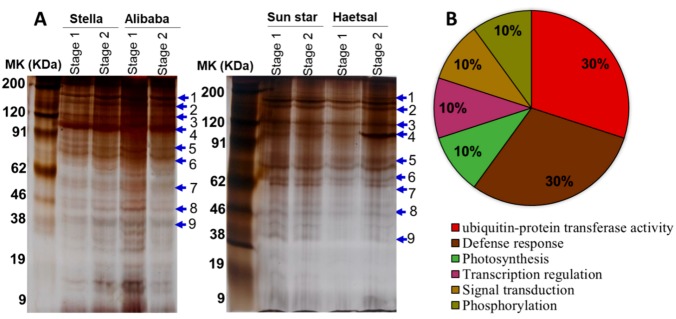
(**A**) Cut rose leaf proteins (20 µg per lane) separated by 1D-SDS-PAGE and stained with silver stain. For each treatment analysis, 1D gels were run in triplicates. Labelled proteins were excised, digested in trypsin, and analyzed by mass spectrometer for subsequent identification and ion search; (**B**) Functional categories of the identified cut rose proteins analyzed bioinformatically by gene ontology. -Resistant and/or -sensitive to aphids; aphid-resistant cultivars are ‘Stella’ and ‘Alibaba’; aphid-sensitive cultivars are ‘Sun star’ and ‘Haetsal’. Stage 1 indicates roses at developed bud stage and stage 2 indicates roses at developed flower stage.

The protein profile was analyzed by first-dimensional gel electrophoresis (SDS-PAGE) followed by the technique of mass spectrometry (Figure 2, Table 1). In general, the comparative 1DE proteome analysis between aphid-resistant cultivars (‘Stella’ and ‘Alibaba’) and aphid-sensitive cultivars (‘Sun star’ and ‘Haetsal’) showed significant variation (Figure 2A). In aphid-resistant cultivars, the number of protein bands was significantly 3–4-fold higher than aphid-sensitive cultivars while no such difference was observed between developmental stages with minor variations. These results were in agreement with the previous results of aphid attacked wheat, however, from their findings it is not clear whether the cultivar used were resistant or sensitive [25,31]. Consequently, our findings suggested that proteins related to resistance or/and sensitivity might play a distinctive role in aphid infestations. The expressed eight to nine protein bands on SDS-PAGE from both aphid-resistant and aphid-sensitive cultivars at both developmental stages (Table 1) were identified by a mass spectrometer (MALDI-TOF MS/MS). Thereafter, the identified proteins were functionally annotated among which 30% were associated to defense response and 30% to ubiquitin transferase activity; the rest were associated with photosynthesis (10%), transcription (10%), signal transduction (10%) and phosphorylation (10%) (Figure 2B).

### 3.2. Proteins Related to Ubiquitin

Plants are consistently exposed to various environmental stresses (biotic/abiotic) such as temperature, salinity, radiation, nutrient deprivations, bugs, aphids, and other insects throughout their life cycle. To ensure their survival they effectively undergo several metabolic changes through various signaling pathways which involve various enzymes, genes, and proteins. Among, signaling pathways ubiquitination is a common practice followed by plants during a stress response. A large number of reports have identified that ubiquitin conjugation as a major regulator to stress response by modulating the activity of stress responsive proteins required for adaptation to stress for example, E3 ubiquitin ligase is involved in regulating drought and salinity stress through abscisic acid signaling [32,33]. Other than stress response ubiquitin is also known to play other roles in plants such as organ development, photomorphogenesis, and hormonal response [34,35]. Consequently, the present study also signifies ubiquitin as one the major classified proteins resistant and/or sensitive to aphid attack in rose cultivars (Table 1). Although the identification of ubiquitin proteins does not give any difference between resistant and sensitive cultivars but nevertheless indicated regulation of stress at two developmental stages viz., bud and flower stage (Stage 1 and 2 respectively).

### 3.3. Proteins Related to the Defense Response

**Figure 3:**
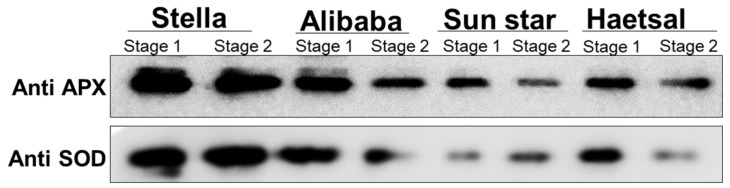
Immunoblot analysis of defense responsive proteins viz., ascorbate peroxidase (APX) and superoxide dismutase (SOD) in cut rose. -Resistant and/or -sensitive to aphids; aphid-resistant cultivars are ‘Stella’ and ‘Alibaba’; aphid-sensitive cultivars are ‘Sun star’ and ‘Haetsal’. Stage 1 indicates roses at developed bud stage and stage 2 indicates roses at developed flower stage.

This functional category comprised proteins related to defense against abiotic or biotic stress, including those involved in the response to oxidative stress. The defense responsive proteins play a major role in alleviating reactive oxygen species (ROS) to hydrogen peroxide (H_2_O_2_) and then water by involving major enzymes like superoxide dismutase (SOD), ascorbate peroxidase (APX), and catalase (CAT) and non-antioxidative enzymes like glutathione and ascorbate [36]. In the present study, we also identified proteins related to defense response like pathogenesis related proteins both in aphid-resistant and/or -sensitive cultivars. The expression of this defense response protein indicates subsequent detoxification of ROS in all cultivars, either resistant or sensitive. The main findings observed in this study showed that resistant cultivars were more likely to detoxify ROS triggered by aphid attack compared to sensitive cultivars as also shown by immunoblots (Figure 3). 

From the immunoblots, it can be easily observed that expression levels of two ROS detoxifying enzymes SOD (superoxide dismutase) and APX (ascorbate peroxidase) was higher in resistant cultivars and lower in sensitive cultivars at both developmental stages. Interestingly we observed a huge difference at developmental stages in aphid sensitive cultivars (Figure 3) and it was observed that expression levels of APX and SOD were high at stage 1 (bud stage) and low at stage 2 (flower stage). This indicates that stage 2 is more prone to oxidative damage by aphid attack compared to stage 1. From these studies it is indicated, that resistant cultivar viz., Stella and Alibaba suitable cultivars for breeding compared to sensitive cultivars.

### 3.4. Proteins Related to Photosynthesis

**Figure 4:**
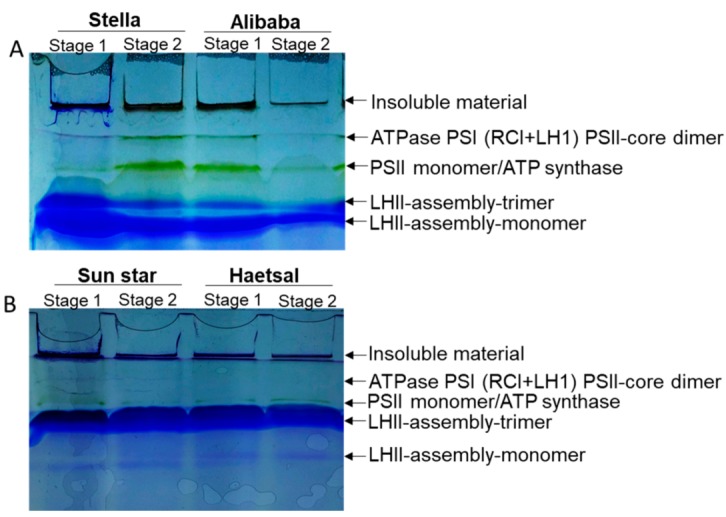
Multiprotein complex proteins (MCPs) of thylakoids/chloroplasts analyzed by blue native polyacrylamide gel electrophoresis (BN-PAGE) in cut rose. Resistant and/or sensitive to aphids; (**A**) aphid-resistant cultivars are ‘Stella’ and ‘Alibaba’; aphid-sensitive cultivars are (**B**) ‘Sun star’ and ‘Haetsal’. Stage 1 indicates roses at developed bud stage and stage 2 indicates roses at developed flower stage. Proteins from all samples were separated on 7–12% gradient gel.

Among other identified proteins in cut roses resistant or sensitive to aphid attack, only 10% of the proteins were classified into photosynthesis. Since photosynthesis is the prime biochemical reaction in plants, we therefore, observed photosynthetic proteins in cut rose cultivars by blue native page (BN-PAGE) (Figure 4). Because from this protein technique we can confirm multiprotein complexes in chloroplasts/thylakoids (photosynthetic organelle) together. From our results, it is very clear that photosynthetic protein bands like ATPase PSI(RC1+LH1)-PSII-core dimer and PSII monomer/ATP synthase are expressed in abundance in aphid -resistant (Stella and Alibaba) rose cultivars. Whereas, these two protein bands can be seen almost negligible in aphid-sensitive cultivars (Sun Star and Haetsal) at both developmental stages with minor changes at Stage 1 of -sensitive cultivar (Sun Star). From these results we can presume that aphid -resistant rose cultivar can undergo redox hemostasis by activating stress response proteins as also shown by our functional categories of proteins. The decreased abundance of these two proteins under abiotic stress have been reported in several plants such as spinach, Arabidopsis, mustard, tomato, Kentucky blue grass and recently in *Physcomitrella patens* [37,38,39,40,41,42,43]. However, those studies either observe only their abundance or pathway for tolerance but our studies described the comparative analysis among -resistant and/or -sensitive cultivars of cut rose which is not yet reported. 

### 3.5. Metabolic Changes

**Figure 5:**
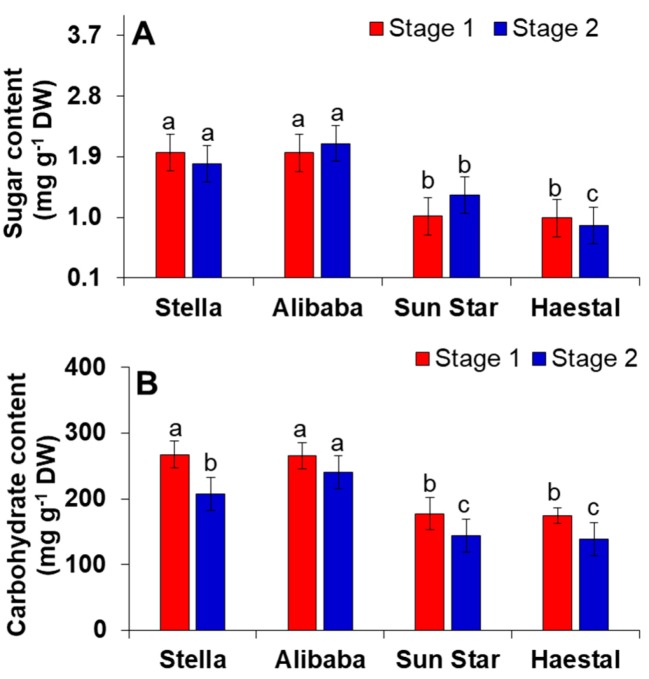
(**A**) Sugar and (**B**) Total carbohydrate content in cut rose. -Resistant and/or -sensitive to aphids; aphid -resistant cultivars are ‘Stella’ and ‘Alibaba’; aphid-sensitive cultivars are ‘Sun star’ and ‘Haetsal’. Stage 1 indicates roses at developed bud stage and stage 2 indicates roses at developed flower stage. Vertical bars indicate ±S.E of the means for *n* = 4. Means denoted by the different letter are significantly different at *p* < 0.05 according to the Tukey’s studentized range test.

Plants undergo several changes during their life cycle due to environmental changes and thus interact with certain phytohormones, proteins, and genes for their growth and development [44,45,46]. Among several metabolic changes, sugar and carbohydrates owe their regulatory function to biotic and abiotic stresses [47,48,49,50]. Our results clearly showed that sugar and carbohydrate levels were significantly highest in -resistant cultivars at stage 1 (bud stage) and slightly lowered at stage 2 (flower stage) (Figure 5) though, was higher than sensitive cultivars. Simultaneously the levels of sugar and carbohydrate were significantly lowest in -sensitive cultivars of cut roses at both developmental stages. Although many reports have suggested that sugars act a prime sensing mechanism to pathogen attack [51,52,53,54]. However, our results indicate that sugars and carbohydrates act a prime inducing precursor to aphid tolerance only in resistant cultivars compared to sensitive cultivars. 

## 4. Conclusions

The present proteome findings showed that ubiquitin proteins regulate stress response (caused by aphid attack) in both aphid-resistant (Stella and Alibaba) and -sensitive (Sun star and Haetsal) cultivars while defense response proteins showed more consistency towards resistant cultivars. This summarizes that aphid–sensitive cultivars are more prone to have oxidative damage compared to aphid–resistant cultivars as shown by the expression levels of two stress responsive proteins such as superoxide dismutase (SOD) and ascorbate peroxidase (APX). Similarly, photosynthetic proteins and metabolic changes (total sugar and carbohydrate content) also gave an overview towards aphid -resistant mechanisms in cut rose cultivars. From the results of photosynthetic proteins particularly multiprotein complex proteins we came into conclusion that sensitive cultivars are more affected towards photosynthetic mechanisms compared to those cultivars which were resistant. Also, this study provided an important information on aphid-resistant and/or -sensitive rose cultivars i.e., which cultivar can be selected for breeding purposes. However, there are still many questions remaining concerning particularly interaction between phloem sap (on which aphids feed themselves) and aphids. Therefore, comprehensive studies on phloem sap following transcriptomics and metabolomics need to be conducted for a detailed mechanism.

## Figures and Tables

**Table 1:** Identification of proteins by MALDI-TOF MS-MS in cut rose cultivars -resistant or -sensitive to aphids.

Band No.	Protein Name	Plant Species	Accession Number	Peptides	Biological Function	Mr Value	Sequence Coverage
	**Resistant cultivars**						
	**Stella**						
1	armadillo/beta-catenin repeat family protein	*Populus trichocarpa*	gi|224066241	METSSVRCLINSISR.F	ubiquitin-protein transferase activity	83,169	12
2	armadillo/beta-catenin repeat family protein	*Populus trichocarpa*	gi|224066241	METSSVRCLINSISR.F	ubiquitin-protein transferase activity	83,169	12
3	Probable protein phosphatase	*Arabidopsis thaliana*	P2C20_ARATH	R. EILHKMK.V	Defense response	31,827	19
4	30S ribosomal protein S11, chloroplastic	*Huperzia lucidula*	RR11_HUPLU	R. GQAVSWSSAGACGSRGTK.K	Photosynthesis	14,124	43
5	Cell division cycle protein 48 homolog	*Capsicum annuum*	CDC48_CAPAN	R. GDTILIKGK.K	Cell division	89,275	14
6	Shikimate kinase 1, chloroplastic	*Oryza sativa*	SK1_ORYSJ	MEAGVGLALQSRAAGFGGSDR.R	Phosphorylation	33,289	54
7	Pathogenesis-related protein 1	*Asparagus officinalis*	AB29G_ARATH	MSSGSWSHEVAVNVAAGRMFK.A	Defense response	160,195	10
	**Alibaba**						
1	armadillo/beta-catenin repeat family protein	*Populus trichocarpa*	gi|224066241	METSSVRCLINSISR.F	ubiquitin-protein transferase activity	83,169	12
2	armadillo/beta-catenin repeat family protein	*Populus trichocarpa*	gi|224066241	METSSVRCLINSISR.F	ubiquitin-protein transferase activity	83,169	12
3	Probable protein phosphatase	*Arabidopsis thaliana*	P2C20_ARATH	R. EILHKMK.V	Defense response	31,827	19
4	30S ribosomal protein S11, chloroplastic	*Huperzia lucidula*	RR11_HUPLU	R. GQAVSWSSAGACGSRGTK.K	Photosynthesis	14,124	43
5	Cell division cycle protein 48 homolog	*Capsicum annuum*	CDC48_CAPAN	R. GDTILIKGK.K	Cell division	89,275	14
6	Shikimate kinase 1, chloroplastic	*Oryza sativa*	SK1_ORYSJ	MEAGVGLALQSRAAGFGGSDR.R	Phosphorylation	33,289	54
7	Pathogenesis-related protein 1	*Asparagus officinalis*	AB29G_ARATH	MSSGSWSHEVAVNVAAGRMFK.A	Defense response	160,195	10
8	1,4-alpha-glucan-branching enzyme	*Solanum tuberosum*	GLGB_SOLTU	K. VSSGASRNK.I	Starch biosynthesis	99,021	11
	**Sensitive cultivars**						
	**Sun Star**						
1	armadillo/beta-catenin repeat family protein	*Populus trichocarpa*	gi|224066241	METSSVRCLINSISR.F	ubiquitin-protein transferase activity	83,169	12
2	armadillo/beta-catenin repeat family protein	*Populus trichocarpa*	gi|224066241	METSSVRCLINSISR.F	ubiquitin-protein transferase activity	83,169	12
3	Probable protein phosphatase	*Arabidopsis thaliana*	P2C20_ARATH	R. EILHKMK.V	Defense response	31,827	19
4	30S ribosomal protein S11, chloroplastic	*Huperzia lucidula*	RR11_HUPLU	R. GQAVSWSSAGACGSRGTK.K	Photosynthesis	14,124	43
5	Cell division cycle protein 48 homolog	*Capsicum annuum*	CDC48_CAPAN	R. GDTILIKGK.K	Cell division	89,275	14
6	Shikimate kinase 1, chloroplastic	*Oryza sativa*	SK1_ORYSJ	MEAGVGLALQSRAAGFGGSDR.R	Phosphorylation	33,289	54
7	Pathogenesis-related protein 1	*Asparagus officinalis*	AB29G_ARATH	MSSGSWSHEVAVNVAAGRMFK.A	Defense response	160,195	10
8	1,4-alpha-glucan-branching enzyme	*Solanum tuberosum*	GLGB_SOLTU	K. VSSGASRNK.I	Starch biosynthesis	99,021	11
	**Haetsal**						
1	armadillo/beta-catenin repeat family protein	*Populus trichocarpa*	gi|224066241	METSSVRCLINSISR.F	ubiquitin-protein transferase activity	83,169	12
2	armadillo/beta-catenin repeat family protein	*Populus trichocarpa*	gi|224066241	METSSVRCLINSISR.F	ubiquitin-protein transferase activity	83,169	12
3	Probable protein phosphatase	*Arabidopsis thaliana*	P2C20_ARATH	R. EILHKMK.V	Defense response	31,827	19
4	30S ribosomal protein S11, chloroplastic	*Huperzia lucidula*	RR11_HUPLU	R. GQAVSWSSAGACGSRGTK.K	Photosynthesis	14,124	43
5	Cell division cycle protein 48 homolog	*Capsicum annuum*	CDC48_CAPAN	R. GDTILIKGK.K	Cell division	89,275	14
6	Shikimate kinase 1, chloroplastic	*Oryza sativa*	SK1_ORYSJ	MEAGVGLALQSRAAGFGGSDR.R	Phosphorylation	33,289	54
7	Pathogenesis-related protein 1	*Asparagus officinalis*	AB29G_ARATH	MSSGSWSHEVAVNVAAGRMFK.A	Defense response	160,195	10
8	1,4-alpha-glucan-branching enzyme	*Solanum tuberosum*	GLGB_SOLTU	K. VSSGASRNK.I	Starch biosynthesis	99,021	11
9	Probable fructokinase-5	*Arabidopsis thaliana*	SCRK5_ARATH	K. APGGAPANVACAITKLGGK.S	Sugar synthesis	34,666	27
